# Axioms for the Boltzmann Distribution

**DOI:** 10.1007/s10701-019-00257-z

**Published:** 2019-05-04

**Authors:** Adam Brandenburger, Kai Steverson

**Affiliations:** 1 Stern School of Business, Tandon School of Engineering, NYU Shanghai, New York University, 44 West 4th Street, New York, NY 10012 USA; 2 Center for Neural Science, New York University, 4 Washington Place, Room 809, New York, NY 10012 USA

**Keywords:** Boltzmann distribution, Equal-probability postulate, Thermodynamics, Axioms

## Abstract

A fundamental postulate of statistical mechanics is that all microstates in an isolated system are equally probable. This postulate, which goes back to Boltzmann, has often been criticized for not having a clear physical foundation. In this note, we provide a derivation of the canonical (Boltzmann) distribution that avoids this postulate. In its place, we impose two axioms with physical interpretations. The first axiom (thermal equilibrium) ensures that, as our system of interest comes into contact with different heat baths, the ranking of states of the system by probability is unchanged. Physically, this axiom is a statement that in thermal equilibrium, population inversions do not arise. The second axiom (energy exchange) requires that, for any heat bath and any probability distribution on states, there is a universe consisting of a system and heat bath that can achieve this distribution. Physically, this axiom is a statement that energy flows between system and heat bath are unrestricted. We show that our two axioms identify the Boltzmann distribution.

## Introduction

The postulates of statistical mechanics have been examined and debated ever since the beginnings of the field in the nineteenth century. A central postulate in equilibrium thermodynamics, put in place by Boltzmann, is that there is equal a priori probability that an isolated system will be found in any one of its microstates which are compatible with the overall constraints placed on the system. In the words of Planck [1], “all microscopic states are equally probable in dynamics”.

The equal-probability assumption has been rationalized in several ways. One can simply appeal to the Laplacian stance of insufficient reason. The observer’s knowledge of the system does not yield a distinction among the microstates, so no distinction can legitimately be introduced via their probabilities of occurrence [2]. Jaynes [3] replaced this assumption with a maximum-entropy principle (a principle of “maximum noncommitment with respect to missing information”) in order to derive the canonical (Boltzmann) distribution in the microcanonical ensemble. Goldstein et al. [4] proved that, for quantum systems, the canonical distribution arises for almost all wave functions of the universe (system plus heat bath). Popescu et al. [5] showed that, even without energy constraints, a “general canonical principle” can be established for quantum systems, under which a system will almost always behave as if the universe is in the equal-probability state.

In this note, we take a different route (for classical systems). We replace the equal-probability postulate with two physically interpretable axioms, which we show characterize the canonical (Boltzmann) distribution.

## Axioms

**Fig. 1:**
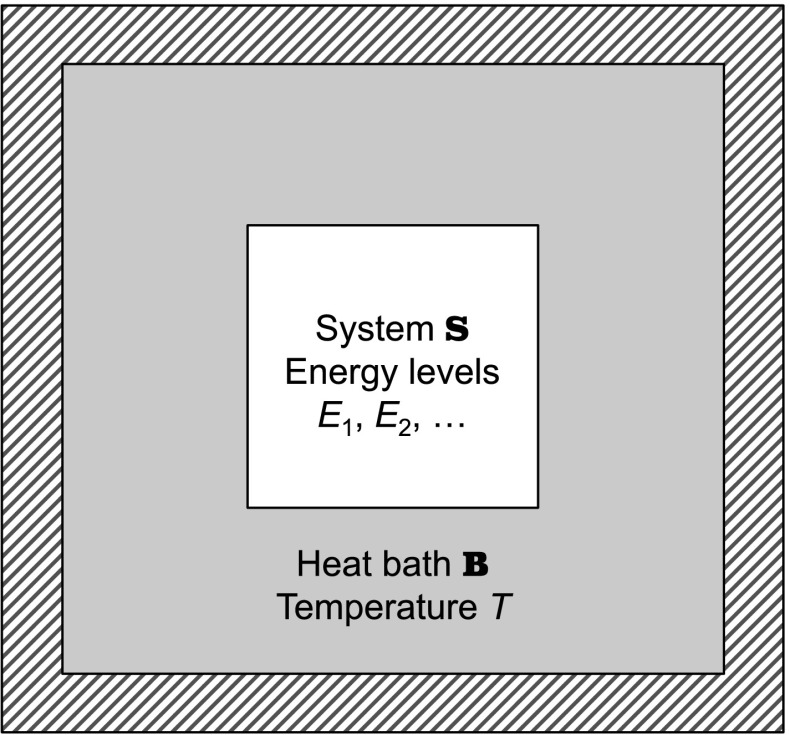
System plus heat bath

In the usual (textbook) derivation, one fixes a heat bath $\mathbb {B}$ at a temperature *T* and a system $\mathbb {S}$ with possible states $s_{i}$, for $i=1,2,\ldots ,n$. The system $\mathbb {S}$ specifies an energy level $E_{i}$ for each state $s_{i}$. (See Fig. 1.) The probability assigned to state $s_{i}$ depends on the system $\mathbb {S}$ and the heat bath $\mathbb {B}$ and can therefore be written as $p_{\mathbb {S}}(s_{i},\mathbb {B})$. One then appeals to the equal-probability postulate to write the ratios of probabilities of states as

1 $$\begin{aligned} \frac{p_{\mathbb {S}}(s_{i};\mathbb {B})}{p_{\mathbb {S}}(s_{j};\mathbb {B})}=\frac{\varOmega _{\mathbb {B}}(E_{\mathrm {total}}-E_{i})}{\varOmega _{\mathbb {B}}(E_{\mathrm {total}}-E_{j})}, \end{aligned}$$

where $E_{\mathrm {total}}$ is the total energy of the composite $\mathbb {S}+\mathbb {B},$ so that $\varOmega _{\mathbb {B}}(E_{\mathrm {total}}-E_{i})$ is then the number of microstates of $\mathbb {B}$. A Taylor expansion of the entropy $S_{\mathbb {B}}(E_{\mathrm {total}}-E_{i})=k\ln \varOmega _{\mathbb {B}}$ of $\mathbb {B}$ (where *k* is the Boltzmann constant), and use of the formula $\partial S_{\mathbb {B}}/\partial E_{\mathrm {total}}=1/T$, yields the Boltmann distribution

2 $$\begin{aligned} p_{\mathbb {S}}(s_{i};\mathbb {B})=\frac{1}{Z}e^{-\frac{E_{i}}{kT}}, \end{aligned}$$

where $Z={\textstyle {\textstyle \varSigma _{j}}}e^{-E_{j}/kT}$ is the partition function (e.g., Mandl [2], pp. 52–56).

Our derivation will also begin with ratios of probabilities, as in Eq. (1), but will not assume the equal-probability postulate. Our axioms are stated over a family $\{\mathbb {S},\mathbb {S}^{\prime },\mathbb {S}^{\prime \prime },\ldots \}$ of systems and a family $\{\mathbb {B},\mathbb {B}^{\prime },\mathbb {B}^{\prime \prime },\ldots \}$ of heat baths. All systems are defined on the same fixed underlying finite set of states $\{s_{1},s_{2},\ldots ,s_{n}\}$.

### Axiom 1

*(Thermal Equilibrium)* Associated with each heat bath $\mathbb {B}$ there is a strictly increasing function $G_{\mathbb {B}}:(0,\infty )\rightarrow (0,\infty )$ such that for any system $\mathbb {S}$ and pair of states $s_{i}$ and $s_{j}$, the ratio equation

3 $$\begin{aligned} G_{\mathbb {B}}\left( \frac{p_{\mathbb {S}}(s_{i};\mathbb {B})}{p_{\mathbb {S}}(s_{j};\mathbb {B})}\right) =G_{\mathbb {\mathbb {B}^{\prime }}}\left( \frac{p_{\mathbb {S}}(s_{i};\mathbb {\mathbb {\mathbb {B}^{\prime }}})}{p_{\mathbb {S}}(s_{j};\mathbb {\mathbb {B}^{\prime }})}\right) \end{aligned}$$

is satisfied.

Our first axiom ensures that the probabilistic ranking of states of the system does not differ with changes in the heat bath. This is physically correct, since we are considering systems in equilibrium and, therefore, population inversions are not possible. If state $s_{i}$ is more likely than another state $s_{j}$, this is because $s_{i}$ is lower energy than $s_{j}$. In thermal equilibrium, the same probabilistic ranking of states will hold whether the heat bath is $\mathbb {B}$ or $\mathbb {B}{}^{\prime }$. Lemma 1 below states this formally. The axiom does allow the actual probability of a state of the system to depend on the particular heat bath $\mathbb {B}$ to which the system is attached. This is the role of the $G_{\mathbb {B}}$-functions. Again, this is physically correct.

### Lemma 1

If $$p_{\mathbb {S}}(s_{i};\mathbb {B})\ge p_{\mathbb {S}}(s_{j};\mathbb {B})$$, then $$p_{\mathbb {S}}(s_{i};\mathbb {\mathbb {B}{}^{\prime }})\ge p_{\mathbb {S}}(s_{j};\mathbb {\mathbb {B}{}^{\prime }})$$.

### Proof

We can write

$$\begin{aligned} \frac{p_{\mathbb {S}}(s_{i};\mathbb {B})}{p_{\mathbb {S}}(s_{j};\mathbb {B})}\ge \frac{p_{\mathbb {S}}(s_{j};\mathbb {\mathbb {B}})}{p_{\mathbb {S}}(s_{i};\mathbb {\mathbb {B}})}, \end{aligned}$$

so that, since $G_{\mathbb {B}}$ is increasing,

$$\begin{aligned} G_{\mathbb {B}}\left( \frac{p_{\mathbb {S}}(s_{i};\mathbb {B})}{p_{\mathbb {S}}(s_{j};\mathbb {B})}\right) \ge G_{\mathbb {\mathbb {\mathbb {B}}}}\left( \frac{p_{\mathbb {S}}(s_{j};\mathbb {\mathbb {B}})}{p_{\mathbb {S}}(s_{i};\mathbb {\mathbb {B}})}\right) . \end{aligned}$$

But, using Eq. (3),

$$\begin{aligned} G_{\mathbb {B}}\left( \frac{p_{\mathbb {S}}(s_{i};\mathbb {\mathbb {B}})}{p_{\mathbb {S}}(s_{j};\mathbb {\mathbb {B}})}\right) =G_{\mathbb {\mathbb {\mathbb {B}{}^{\prime }}}}\left( \frac{p_{\mathbb {S}}(s_{i};\mathbb {\mathbb {B}{}^{\prime }})}{p_{\mathbb {S}}(s_{j};\mathbb {\mathbb {B}{}^{\prime }})}\right) \text{ and } G_{\mathbb {\mathbb {\mathbb {B}}}}\left( \frac{p_{\mathbb {S}}(s_{j};\mathbb {\mathbb {B}})}{p_{\mathbb {S}}(s_{i};\mathbb {\mathbb {B}})}\right) =G_{\mathbb {\mathbb {\mathbb {B}{}^{\prime }}}}\left( \frac{p_{\mathbb {S}}(s_{j};\mathbb {\mathbb {B}{}^{\prime }})}{p_{\mathbb {S}}(s_{i};\mathbb {\mathbb {B}{}^{\prime }})}\right) , \end{aligned}$$

and, therefore,

$$\begin{aligned} G_{\mathbb {\mathbb {\mathbb {B}{}^{\prime }}}}\left( \frac{p_{\mathbb {S}}(s_{i};\mathbb {\mathbb {B}{}^{\prime }})}{p_{\mathbb {S}}(s_{j};\mathbb {\mathbb {B}{}^{\prime }})}\right) \ge G_{\mathbb {\mathbb {\mathbb {B}{}^{\prime }}}}\left( \frac{p_{\mathbb {S}}(s_{j};\mathbb {\mathbb {B}{}^{\prime }})}{p_{\mathbb {S}}(s_{i};\mathbb {\mathbb {B}{}^{\prime }})}\right) , \end{aligned}$$

from which, since $G_{\mathbb {\mathbb {\mathbb {B}{}^{\prime }}}}$ is increasing,

$$\begin{aligned} \frac{p_{\mathbb {S}}(s_{i};\mathbb {\mathbb {B}{}^{\prime }})}{p_{\mathbb {S}}(s_{j};\mathbb {\mathbb {B}{}^{\prime }})}\ge \frac{p_{\mathbb {S}}(s_{j};\mathbb {\mathbb {B}{}^{\prime }})}{p_{\mathbb {S}}(s_{i};\mathbb {\mathbb {B}{}^{\prime }})}, \end{aligned}$$

or $p_{\mathbb {S}}(s_{i};\mathbb {\mathbb {B}{}^{\prime }})\ge p_{\mathbb {S}}(s_{j};\mathbb {\mathbb {B}{}^{\prime }})$, as required. $\square$

Our second axiom is designed to capture the fact that a heat bath $\mathbb {B}$ is very large compared with a system $\mathbb {S}$, so that any energy flows are possible between the two at the given temperature of the bath. We say this formally by fixing a heat bath $\mathbb {B}$ and a probability distribution on the states $\{s_{1},s_{2},\ldots ,s_{n}\}$. We then say that we can attach a system $\mathbb {S}$ to $\mathbb {B}$ so that the desired probabilities are obtained. Physically, we know we can do this. Indeed, Eq. (2) for the Boltzmann distribution tells us there are energy levels $E_{i}$, for $i=1,2,\ldots ,n$, that yield the probabilities in question. (If $\lambda _{i}$ is the probability of state *i*, then we set $E_{i}=-kT\ln \lambda _{i}$.) So, we attach a system $\mathbb {S}$ with these energy levels to the heat bath $\mathbb {B}$. Since $\mathbb {B}$ is very large compared with $\mathbb {S}$, we can always do this at the prevailing temperature *T*. Here is the formal statement. (We assume that $\lambda$ has full support, i.e, that $\lambda _{i}>0$ for all *i*. This guarantees that all ratios of probabilities are well-defined.)

### Axiom 2

*(Energy Exchange)* For any heat bath $\mathbb {B}$ and any full-support probability distribution $\lambda =(\lambda _{1},\lambda _{2},\ldots ,\lambda _{n})$ on $\{s_{1},s_{2},\ldots ,s_{n}\}$, there is a system $\mathbb {S}$ such that $p_{\mathbb {S}}(\cdot ;\mathbb {B})=\lambda$.

## Result

We can now state our result, which is an axiomatic derivation of the Boltzmann distribution.

### Theorem 1

Suppose Axioms 1 and 2 are satisfied. Then there are functions $T:\{\mathbb {B},\mathbb {B}^{\prime },\mathbb {B}^{\prime \prime },\ldots \}\rightarrow (0,\infty )$ and $E:\{s_{1},s_{2},\ldots ,s_{n}\}\times \{\mathbb {S},\mathbb {S}^{\prime },\mathbb {S}^{\prime \prime },\ldots \}\rightarrow (0,\infty )$ such that for each heat bath $\mathbb {B}$ and system $\mathbb {S}$, and for each $i=1,2,\ldots ,n$,

4 $$\begin{aligned} p_{\mathbb {S}}(s_{i};\mathbb {B})=\frac{1}{Z(\mathbb {B},\mathbb {S})}e^{-\frac{E(s_{i},\mathbb {S})}{T(\mathbb {B})}}, \end{aligned}$$

where $$Z(\mathbb {B},\mathbb {S})={\textstyle {\textstyle \varSigma _{j}}}e^{-E(s_{j},\mathbb {S})/T(\mathbb {B})}$$.

Equation (4) is the Boltzmann distribution, with temperature $T(\cdot )$ (as a function of the heat bath) and energy levels $E(s_{1},\cdot ),E(s_{2},\mathbb {\cdot }),\ldots ,E(s_{n},\mathbb {\cdot })$ (as a function of the system). (We get $k=1$ since temperature and energy are not measured in physical units here). Notice that only positive temperatures are possible under our treatment. This makes sense, since we have assumed thermal equilibrium, and negative temperatures can arise only in systems which are (temporarily) out of equilibrium (e.g., Braun et al. [6]). Also, as expected in an abstract treatment, the fundamental quantity that emerges is $E(\cdot ,\cdot )/T(\cdot )$, namely, entropy. We can be more precise about this last point by establishing the uniqueness properties of the functions *T* and *E* that represent a given heat bath and system.

### Theorem 2

Assume that, for each heat bath $\mathbb {B}$, it is not the case that all states have equal probability. Suppose a system $\mathbb {S}$ satisfies Eq. (4) with functions *E* and *T*. Then $\mathbb {S}$ satisfies Eq. (4) with functions $\widetilde{E}$ and $\widetilde{T}$ if and only if there are real numbers $\alpha >0$ and $\beta$ such that

$$\begin{aligned} E(s_{i},\mathbb {S})= & {} \alpha \widetilde{E}(s_{i},\mathbb {S})\,+\,\beta \hbox { for all states }s_{i},\\ T(\mathbb {B})= & {} \alpha \widetilde{T}(\mathbb {B})\hbox { for all heat baths }\mathbb {B}. \end{aligned}$$

(Physically speaking, the equal-probability case ruled out is that of infinite temperature.) Notice that the scaling factor for *T* is the same as the multiplicative factor in the affine transformation of *E*. It follows that, while the ratios $E\left( \cdot ,\cdot \right) /T(\cdot )$ are not unique, the differences between these ratios, i.e., the entropy differences

$$\begin{aligned} \frac{E(s_{i},\mathbb {S})-E(s_{j},\mathbb {S})}{T\left( \mathbb {B}\right) } \end{aligned}$$

between states, are unique. Again, we expect this on physical grounds.

## Summary

We have shown that two physically interpretable axioms can replace the traditional equal-probability postulate of equilibrium thermodynamics. The first axiom is an abstraction of the notion that the probabilistic ranking of states is the same across systems in equilibrium. The second axiom is an abstraction of the notion that all energy flows are possible between the system in question and a heat bath to which it is attached, at the given temperature of the bath. Together, these two axioms characterize the Boltzmann distribution. That is, we establish that the axioms identify the Boltzmann distribution—and the converse that the Boltzmann distribution satisfies the axioms.

Two extensions of this work would be interesting. The first extension would be to quantum systems, to see if our characterization goes through and to compare the resulting analysis with those of Goldstein et al. [4] and Popescu et al. [5]. A second extension would be to continuous probability distributions, where new mathematical issues may arise.

## Proof of Theorem 1

We choose heat bath $\mathbb {B}$ as a reference point. Since $G_{\mathbb {B}}$ is strictly increasing, it is invertible. Therefore, for any (other) heat bath $\mathbb {B^{\prime }}$, we can define a function $H_{\mathbb {\mathbb {B^{\prime }}}}:\left( 0,\infty \right) \rightarrow \left( 0,\infty \right)$ by

$$\begin{aligned} H_{\mathbb {\mathbb {B^{\prime }}}}\left( t\right) =G_{\mathbb {B}}^{-1}(G_{\mathbb {\mathbb {B^{\prime }}}}(t)). \end{aligned}$$

By Axiom 1, we have that for any system $\mathbb {S}$ and pair of states *r*, *s*,

$$\begin{aligned} G_{\mathbb {\mathbb {B^{\prime }}}}\left( \frac{p_{\mathbb {S}}(r;\mathbb {\mathbb {B^{\prime }}})}{p_{\mathbb {S}}(s;\mathbb {\mathbb {B^{\prime }}})}\right) =G_{\mathbb {B}}\left( \frac{p_{\mathbb {S}}(r;\mathbb {B})}{p_{\mathbb {S}}(s;\mathbb {B})}\right) , \end{aligned}$$

so that

5 $$\begin{aligned} H_{\mathbb {\mathbb {\mathbb {B^{\prime }}}}}\left( \frac{p_{\mathbb {S}}(r;\mathbb {\mathbb {B^{\prime }}})}{p_{\mathbb {S}}(s;\mathbb {\mathbb {B^{\prime }}})}\right) =\frac{p_{\mathbb {S}}(r;\mathbb {B})}{p_{\mathbb {S}}(s;\mathbb {B})}. \end{aligned}$$

It follows that for any triplet of states $s_{i},s_{j},s_{k}$,

$$\begin{aligned} H_{\mathbb {\mathbb {B^{\prime }}}}\left( \frac{p_{\mathbb {S}}(s_{i};\mathbb {\mathbb {B^{\prime }}})}{p_{\mathbb {S}}(s_{j};\mathbb {\mathbb {B^{\prime }}})}\right) \times H_{\mathbb {\mathbb {B^{\prime }}}}\left( \frac{p_{\mathbb {S}}(s_{j};\mathbb {\mathbb {B^{\prime }}})}{p_{\mathbb {S}}(s_{k};\mathbb {\mathbb {B^{\prime }}})}\right) =\frac{p_{\mathbb {S}}(s_{i};\mathbb {B})}{p_{\mathbb {S}}(s_{j};\mathbb {B})}\times \frac{p_{\mathbb {S}}(s_{j};\mathbb {B})}{p_{\mathbb {S}}(s_{k};\mathbb {B})}=\frac{p_{\mathbb {S}}(s_{i};\mathbb {B})}{p_{\mathbb {S}}(s_{k};\mathbb {B})}. \end{aligned}$$

We can also write

$$\begin{aligned} H_{\mathbb {\mathbb {\mathbb {B^{\prime }}}}}\left( \frac{p_{\mathbb {S}}(s_{i};\mathbb {\mathbb {B^{\prime }}})}{p_{\mathbb {S}}(s_{k};\mathbb {\mathbb {B^{\prime }}})}\right) =\frac{p_{\mathbb {S}}(s_{i};\mathbb {B})}{p_{\mathbb {S}}(s_{k};\mathbb {B})}. \end{aligned}$$

Putting these two equations together yields

6 $$\begin{aligned} H_{\mathbb {\mathbb {B^{\prime }}}}\left( \frac{p_{\mathbb {S}}(s_{i};\mathbb {\mathbb {B^{\prime }}})}{p_{\mathbb {S}}(s_{j};\mathbb {\mathbb {B^{\prime }}})}\right) \times H_{\mathbb {\mathbb {B^{\prime }}}}\left( \frac{p_{\mathbb {S}}(s_{j};\mathbb {\mathbb {B^{\prime }}})}{p_{\mathbb {S}}(s_{k};\mathbb {\mathbb {B^{\prime }}})}\right) =H_{\mathbb {\mathbb {\mathbb {B^{\prime }}}}}\left( \frac{p_{\mathbb {S}}(s_{i};\mathbb {\mathbb {B^{\prime }}})}{p_{\mathbb {S}}(s_{k};\mathbb {\mathbb {B^{\prime }}})}\right) . \end{aligned}$$

We want to turn Eq. (6) into the Cauchy functional equation. To do so, we need an intermediate result. (This result assumes that there are at least three states. The case of two states is treated later.)

### Lemma 2

For any $t,u\in (0,\infty )$, we can choose states $s_{i},s_{j},s_{k}$ and a full-support probability distribution $\lambda$ on $\{s_{1},s_{2},\ldots ,s_{n}\}$ so that

7 $$\begin{aligned} \frac{\lambda (s_{i})}{\lambda (s_{j})}=t\hbox { and }\frac{\lambda (s_{j})}{\lambda (s_{k})}=u. \end{aligned}$$

### Proof

Choose three distinct states $s_{i},s_{j},s_{k}$, and set

$$\begin{aligned} \lambda (s_{i})= & {} \frac{tuv}{1+u+tu},\\ \lambda (s_{j})= & {} \frac{uv}{1+u+tu},\\ \lambda (s_{k})= & {} \frac{v}{1+u+tu}, \end{aligned}$$

where $v=1$ if $n=3$ (there are three states in total) and $v=\tfrac{1}{2}$ if $n>3$. Also, if $n>3$, set

$$\begin{aligned} \lambda \left( s\right) =\frac{1}{2(n-3)}, \end{aligned}$$

for all $s\ne s_{i},s_{j},s_{k}$. It is easy to check that $\lambda$ has full support and that Equation (7) is satisfied. Also, if $n=3$, then

$$\begin{aligned} \sum _{s}\lambda (s)=\frac{tuv+uv+v}{1+u+tu}=v=1, \end{aligned}$$

and if $$n>3$$,

$$\begin{aligned} \sum _{s}\lambda (s)=\sum _{s\ne s_{i},s_{j},s_{k}}\frac{1}{2(n-3)}+\frac{tuv+uv+v}{1+u+tu}=\frac{1}{2}+v=1, \end{aligned}$$

so that $\lambda$ is a well-defined probability distribution on the states. $\square$

By Axioms 1 and 2, there is a system $\mathbb {S}$ so that Eq. (3) is satisfied and $p_{\mathbb {S}}(\cdot ;\mathbb {\mathbb {B^{\prime }}})=\lambda$. But then $p_{\mathbb {S}}(\cdot ;\mathbb {\mathbb {B^{\prime }}})$ also satisfies Eq. (6), and, therefore, using Eq. (7), we obtain

8 $$\begin{aligned} H_{\mathbb {\mathbb {\mathbb {B^{\prime }}}}}(t)\times H_{\mathbb {\mathbb {\mathbb {B^{\prime }}}}}(u)=H_{\mathbb {\mathbb {\mathbb {B^{\prime }}}}}(tu), \end{aligned}$$

for any $t,u\in (0,\infty )$. Moreover, the functions $G_{\mathbb {B}}$ and $G_{\mathbb {\mathbb {\mathbb {B^{\prime }}}}}$ are increasing and therefore have at most a countable number of discontinuities, from which it follows that $H_{\mathbb {\mathbb {B^{\prime }}}}$ can have at most a countable number of discontinuities. This allows us to apply a version of the Cauchy functional theorem (see Appendix C) to Eq. (8), to conclude that there exists a function $T:\{\mathbb {B},\mathbb {B}^{\prime },\mathbb {B}^{\prime \prime },\ldots \}\rightarrow (0,\infty )$ such that

9 $$\begin{aligned} H_{\mathbb {\mathbb {B}^{\prime }}}(t)=t^{T(\mathbb {\mathbb {B}^{\prime }})}. \end{aligned}$$

Note that $T(\mathbb {B})=1$ (this is why we called $\mathbb {B}$ a reference point). Also, from Eq. (9) we get

$$\begin{aligned} G_{\mathbb {\mathbb {B}^{\prime }}}(t)=G_{\mathbb {B}}(t^{T(\mathbb {\mathbb {B}^{\prime }})}). \end{aligned}$$

Since $G_{\mathbb {\mathbb {B}^{\prime }}}$ and $G_{\mathbb {B}}$ are both strictly increasing, it follows that $T(\mathbb {\mathbb {B}^{\prime }})>0$ for all heat baths $\mathbb {\mathbb {B}^{\prime }}$. Next, using Eqs. (9) in (5), we find

$$\begin{aligned} \frac{p_{\mathbb {S}}(r;\mathbb {\mathbb {B^{\prime }}})}{p_{\mathbb {S}}(s;\mathbb {\mathbb {B^{\prime }}})}=\left( \frac{p_{\mathbb {S}}(r;\mathbb {\mathbb {B}})}{p_{\mathbb {S}}(s;\mathbb {\mathbb {B}})}\right) ^{\frac{1}{T(\mathbb {\mathbb {B}^{\prime }})}}. \end{aligned}$$

Summing over all states *r* yields

$$\begin{aligned} \frac{1}{p_{\mathbb {S}}(s;\mathbb {\mathbb {B^{\prime }}})}=\sum _{r}\left( \frac{p_{\mathbb {S}}(r;\mathbb {\mathbb {B}})}{p_{\mathbb {S}}(s;\mathbb {\mathbb {B}})}\right) ^{\frac{1}{T(\mathbb {\mathbb {B}^{\prime }})}}, \end{aligned}$$

which we can invert to get

10 $$\begin{aligned} p_{\mathbb {S}}(s;\mathbb {\mathbb {B^{\prime }}})=\dfrac{p_{\mathbb {S}}(s;\mathbb {\mathbb {B}}){}^{\frac{1}{T(\mathbb {\mathbb {\mathbb {B}^{\prime }}})}}}{\sum _{r}p_{\mathbb {S}}(r;\mathbb {\mathbb {B}})^{\frac{1}{T(\mathbb {\mathbb {B}^{\prime }})}}}. \end{aligned}$$

Finally, to make Eq. (10) into the Boltzmann distribution, define $E:\{s_{1},s_{2},\ldots ,s_{n}\}\times \{\mathbb {S},\mathbb {S}^{\prime },\mathbb {S}^{\prime \prime },\ldots \}\rightarrow (0,\infty )$ by $E(r,\mathbb {S})=-\ln p_{\mathbb {S}}(r;\mathbb {\mathbb {B}})$ for each state *r*.

This completes the proof of Theorem 1, except for the case of two states. (The case of one state is trivial.) Here, we define the function *T* directly, by requiring it to give the solution to each equation

$$\begin{aligned} \left( \frac{p_{\mathbb {S}}(s_{1};\mathbb {\mathbb {B^{\prime }}})}{p_{\mathbb {S}}(s_{2};\mathbb {\mathbb {B^{\prime }}})}\right) ^{T(\mathbb {B^{\prime }})}=\frac{p_{\mathbb {S}}(s_{1};\mathbb {\mathbb {B}})}{p_{\mathbb {S}}(s_{2};\mathbb {\mathbb {B}})}, \end{aligned}$$

as we vary the heat bath $\mathbb {B^{\prime }}$. We can argue similarly to Lemma 1 to see that $p_{\mathbb {S}}(s_{1};\mathbb {\mathbb {B^{\prime }}})\ge p_{\mathbb {S}}(s_{2};\mathbb {\mathbb {B^{\prime }}})$ if and only if $p_{\mathbb {S}}(s_{1};\mathbb {\mathbb {B}})\ge p_{\mathbb {S}}(s_{2};\mathbb {\mathbb {B}})$. It follows that we will get $T(\mathbb {\mathbb {B^{\prime }}})>0$ for all $\mathbb {\mathbb {B^{\prime }}}$, as required.

We should also establish that our axioms identify the Boltzmann distribution and not some subfamily of this distribution. To show this, start by supposing that Eq. (4) holds. Define $G_{\mathbb {B^{\prime }}}:\left( 0,\infty \right) \rightarrow \left( 0,\infty \right)$ by $G_{\mathbb {B^{\prime }}}\left( t\right) =t^{T(\mathbb {B^{\prime }})}$. Then for any system $\mathbb {S}$ and pair of states *r*, *s*, we can write

$$\begin{aligned} G_{\mathbb {B^{\prime }}}\left( \frac{p_{\mathbb {S}}(r;\mathbb {\mathbb {B^{\prime }}})}{p_{\mathbb {S}}(s;\mathbb {\mathbb {B^{\prime }}})}\right) =G_{\mathbb {B^{\prime }}}\left( e^{\frac{E(s,\mathbb {S})-E(r,\mathbb {S})}{T(\mathbb {B^{\prime }})}}\right) =e^{E(s,\mathbb {S})-E(r,\mathbb {S})}. \end{aligned}$$

Since the right-hand side is independent of $\mathbb {B^{\prime }}$, we see that Eq. (3) is satisfied, which establishes Axiom 1. For Axiom 2, fix a heat bath $\mathbb {B^{\prime }}$ and a full-support probability distribution $\lambda$ on the states. Let $T(\mathbb {B}{}^{\prime })$ be arbitrary and set $E(s_{i},\mathbb {S})=-kT(\mathbb {B}{}^{\prime })\ln \lambda _{i}$ for each *i*. Then $p_{\mathbb {S}}(\cdot ;\mathbb {\mathbb {B^{\prime }}})=\lambda$, as required.

## Proof of Theorem 2

Suppose a system $\mathbb {S}$ satisfies Eq. (4) for two pairs of functions *E*, *T* and $\widetilde{E},\widetilde{T}$. Equation (4) implies that for any states $s_{1},s_{2},s$,

$$\begin{aligned} \frac{E(s_{2},\mathbb {S})-E(s_{1},\mathbb {S})}{E(s_{2},\mathbb {S})-E(s,\mathbb {S})}=\left( \ln \frac{p_{\mathbb {S}}(s_{1};\mathbb {\mathbb {B}})}{p_{\mathbb {S}}(s_{2};\mathbb {\mathbb {B}})}\right) \left( \ln \frac{p_{\mathbb {S}}(s;\mathbb {\mathbb {B}})}{p_{\mathbb {S}}(s_{2};\mathbb {\mathbb {B}})}\right) ^{-1}=\frac{\widetilde{E}(s_{2},\mathbb {S})-\widetilde{E}(s_{1},\mathbb {S})}{\widetilde{E}(s_{2},\mathbb {S})-\widetilde{E}(s,\mathbb {S})}. \end{aligned}$$

Rearranging gives

$$\begin{aligned}&(E(s_{2},\mathbb {S})-E(s_{1},\mathbb {S}))\times (\widetilde{E}(s_{2},\mathbb {S})- \widetilde{E}(s,\mathbb {S}))\\&\quad =(E(s_{2},\mathbb {S})-E(s,\mathbb {S}))\times (\widetilde{E}(s_{2},\mathbb {S})-\widetilde{E}(s_{1},\mathbb {S})), \end{aligned}$$

from which,

$$\begin{aligned} E(s,\mathbb {S})=E(s_{2},\mathbb {S})-\frac{E(s_{2},\mathbb {S})-E(s_{1},\mathbb {S})}{\widetilde{E}(s_{2},\mathbb {S})-\widetilde{E}(s_{1},\mathbb {S})}\times (\widetilde{E}(s_{2},\mathbb {S})-\widetilde{E}(s,\mathbb {S})), \end{aligned}$$

and, therefore,

$$\begin{aligned} E(s,\mathbb {S})= & {} \frac{E(s_{2},\mathbb {S})-E(s_{1},\mathbb {S})}{\widetilde{E}(s_{2},\mathbb {S})-\widetilde{E}(s_{1},\mathbb {S})} \times \widetilde{E}(s,\mathbb {S})+E(s_{2},\mathbb {S})\\&- \frac{E(s_{2},\mathbb {S})-E(s_{1},\mathbb {S})}{\widetilde{E}(s_{2},\mathbb {S})-\widetilde{E}(s_{1},\mathbb {S})}\times \widetilde{E}(s_{2},\mathbb {S}). \end{aligned}$$

Now set

$$\begin{aligned} \alpha =\frac{E(s_{2},\mathbb {S})-E(s_{1},\mathbb {S})}{\widetilde{E}(s_{2},\mathbb {S})-\widetilde{E}(s_{1},\mathbb {S})}\text { and }\beta =E(s_{2},\mathbb {S})-\frac{E(s_{2},\mathbb {S})-E(s_{1},\mathbb {S})}{\widetilde{E}(s_{2},\mathbb {S})-\widetilde{E}(s_{1},\mathbb {S})}\times \widetilde{E}(s_{2},\mathbb {S}). \end{aligned}$$

By assumption, there are states $s_{1},s_{2}$ such that $p_{\mathbb {S}}(s_{1};\mathbb {\mathbb {B}})>p_{\mathbb {S}}(s_{2};\mathbb {\mathbb {B}})$. (There is no loss of generality in labeling these two states this way.) It follows that $\alpha >0$.

Next observe that, for any heat bath $\mathbb {\mathbb {B^{\prime }}}$,

$$\begin{aligned} \ln \left( \frac{p_{\mathbb {S}}(s_{2};\mathbb {\mathbb {\mathbb {B^{\prime }}}})}{p_{\mathbb {S}}(s_{1};\mathbb {\mathbb {\mathbb {B^{\prime }}}})}\right) =\frac{E(s_{1},\mathbb {S})-E(s_{2},\mathbb {S})}{T(\mathbb {\mathbb {B^{\prime }}})}=\frac{\widetilde{E}(s_{1},\mathbb {S})-\widetilde{E}(s_{2},\mathbb {S})}{\widetilde{T}(\mathbb {\mathbb {B^{\prime }}})}, \end{aligned}$$

from which, using the relationship between *E* and $\widetilde{E}$, we get

$$\begin{aligned} \frac{\alpha \widetilde{E}(s_{1},\mathbb {S})-\alpha \widetilde{E}(s_{2},\mathbb {S})}{T(\mathbb {\mathbb {B^{\prime }}})}=\frac{\widetilde{E}(s_{1},\mathbb {S})-\widetilde{E}(s_{2},\mathbb {S})}{\widetilde{T}(\mathbb {\mathbb {B^{\prime }}})}. \end{aligned}$$

By assumption, $p_{\mathbb {S}}(s_{1};\mathbb {\mathbb {B}})\ne p_{\mathbb {S}}(s_{2};\mathbb {\mathbb {B}})$. (Again, there is no loss of generality in using the state labels $s_{1}$ and $s_{2}$.) It follows that $\widetilde{E}(s_{1},\mathbb {S})\ne \widetilde{E}(s_{2},\mathbb {S})$ and, therefore, $T(\mathbb {\mathbb {\mathbb {B^{\prime }}}})=\alpha \widetilde{T}(\mathbb {\mathbb {\mathbb {B^{\prime }}}})$, as claimed. This completes the proof of the forward direction of Theorem 2.

For the reverse direction, suppose that a system $\mathbb {S}$ satisfies Eq. (4) for the functions *E* and *T*, and let $\alpha >0$ and $\beta$ be real numbers. Equation (4) then yields, for any heat bath $\mathbb {\mathbb {\mathbb {B^{\prime }}}}$,

$$\begin{aligned} p_{\mathbb {S}}(s;\mathbb {\mathbb {\mathbb {\mathbb {\mathbb {B^{\prime }}}}}})= & {} \frac{e^{-\frac{E(s,\mathbb {S})}{T(\mathbb {\mathbb {\mathbb {\mathbb {B^{\prime }}}}})}}}{\sum _{j}e^{-\frac{E(s,\mathbb {S})}{T(\mathbb {\mathbb {\mathbb {\mathbb {B^{\prime }}}}})}}}=\frac{e^{-\frac{\alpha E(s,\mathbb {S})}{\alpha T(\mathbb {\mathbb {\mathbb {\mathbb {B^{\prime }}}}})}}}{\sum _{j}e^{-\frac{\alpha E(s,\mathbb {S})}{\alpha T(\mathbb {\mathbb {\mathbb {\mathbb {B^{\prime }}}}})}}}\\= & {} \frac{e^{-\frac{\beta }{\alpha T(\mathbb {\mathbb {\mathbb {\mathbb {B^{\prime }}}}})}}e^{-\frac{\alpha E(s,\mathbb {S})}{\alpha T(\mathbb {\mathbb {\mathbb {\mathbb {B^{\prime }}}}})}}}{e^{-\frac{\beta }{\alpha T(\mathbb {\mathbb {\mathbb {\mathbb {B^{\prime }}}}})}}\sum _{j}e^{-\frac{\alpha E(s,\mathbb {S})}{\alpha T(\mathbb {\mathbb {\mathbb {\mathbb {B^{\prime }}}}})}}}=\frac{e^{-\frac{\alpha E(s,\mathbb {S})+\beta }{\alpha T(\mathbb {\mathbb {\mathbb {\mathbb {B^{\prime }}}}})}}}{\sum _{j}e^{-\frac{\alpha E(s,\mathbb {S})+\beta }{\alpha T(\mathbb {\mathbb {\mathbb {\mathbb {B^{\prime }}}}})}}}, \end{aligned}$$

from which we see that the system $\mathbb {S}$ satisfies Eq. (4) for the functions $\alpha E+\beta$ and $\alpha T$, as we needed to show.

To prove that entropy differences are unique, as asserted after the statement of Theorem 2, first suppose that a system $\mathbb {S}$ satisfies Eq. (4) for the functions *E*, *T* and $\widetilde{E},\widetilde{T}$. Theorem 2 tells us that there are real numbers $\alpha >0$ and $\beta$ such that $E(\cdot ,\mathbb {S})=\alpha \widetilde{E}(\cdot ,\mathbb {S})+\beta$ and $T(\cdot )=\alpha \widetilde{T}(\cdot )$. It follows that for any pair of states $s_{i},s_{j}$, and any heat bath $\mathbb {\mathbb {B^{\prime }}}$,

$$\begin{aligned} \frac{E(s_{i},\mathbb {S})}{T(\mathbb {\mathbb {B^{\prime }}})}-\frac{E(s_{j},\mathbb {S})}{T(\mathbb {\mathbb {B^{\prime }}})}=\frac{\alpha \widetilde{E}(s_{i},\mathbb {S})+\beta }{\alpha \widetilde{T}(\mathbb {\mathbb {B^{\prime }}})}-\frac{\alpha \widetilde{E}(s_{j},\mathbb {S})+\beta }{\alpha \widetilde{T}(\mathbb {\mathbb {B^{\prime }}})}=\frac{\widetilde{E}(s_{i},\mathbb {S})}{\widetilde{T}(\mathbb {\mathbb {B^{\prime }}})}-\frac{\widetilde{E}(s_{j},\mathbb {S})}{\widetilde{T}(\mathbb {\mathbb {B^{\prime }}})}, \end{aligned}$$

as claimed. Conversely, suppose a system $\mathbb {S}$ satisfies Eq. (4) for the functions *E*, *T*, and there exist functions $\widetilde{E},\widetilde{T}$ such that

$$\begin{aligned} \frac{\widetilde{E}(s_{i},\mathbb {S})}{\widetilde{T}(\mathbb {\mathbb {B^{\prime }}})}-\frac{\widetilde{E}(s_{j},\mathbb {S})}{\widetilde{T}(\mathbb {\mathbb {B^{\prime }}})}=\frac{E(s_{i},\mathbb {S})}{T(\mathbb {\mathbb {B^{\prime }}})}-\frac{E(s_{j},\mathbb {S})}{T(\mathbb {\mathbb {B^{\prime }}})}. \end{aligned}$$

This says that for each heat bath $\mathbb {\mathbb {B^{\prime }}}$, there is a number $\gamma _{\mathbb {\mathbb {B^{\prime }}}}$ such that

$$\begin{aligned} \frac{\widetilde{E}(s_{i},\mathbb {S})}{\widetilde{T}(\mathbb {\mathbb {B^{\prime }}})}=\frac{E(s_{i},\mathbb {S})}{T(\mathbb {\mathbb {B^{\prime }}})}+\gamma _{\mathbb {\mathbb {B^{\prime }}}}. \end{aligned}$$

It follows that

$$\begin{aligned} \frac{e^{-\frac{\widetilde{E}(s_{i},\mathbb {S})}{\widetilde{T}(\mathbb {\mathbb {B^{\prime }}})}}}{\sum _{j} e^{-\frac{\widetilde{E}(s_{j},\mathbb {S})}{\widetilde{T} (\mathbb {\mathbb {B^{\prime }}})}}}= & {} \frac{e^{-\frac{E(s_{i},\mathbb {S})}{T(\mathbb {\mathbb {B^{\prime }}})}- \gamma _{\mathbb {\mathbb {B^{\prime }}}}}}{\sum _{j}e^{-\frac{E(s_{j}, \mathbb {S})}{T(\mathbb {\mathbb {B^{\prime }}})}- \gamma _{\mathbb {\mathbb {B^{\prime }}}}}} \\= & {} \frac{e^{-\gamma _{\mathbb {\mathbb {B^{\prime }}}}}e^{-\frac{E(s_{i}, \mathbb {S})}{T(\mathbb {\mathbb {B^{\prime }}})}}}{e^{-\gamma _{\mathbb {\mathbb {B^{\prime }}}}} \sum _{j}e^{-\frac{E(s_{j},\mathbb {S})}{T(\mathbb {\mathbb {B^{\prime }}})}}}\\= & {} \frac{e^{-\frac{E(s_{i},\mathbb {S})}{T(\mathbb {\mathbb {B^{\prime }}})}}}{\sum _{j}e^{-\frac{E(s_{j},\mathbb {S})}{T(\mathbb {\mathbb {B^{\prime }}})}}} =p_{\mathbb {S}}(s_{i};\mathbb {\mathbb {B^{\prime }}}), \end{aligned}$$

from which we see that the system $\mathbb {S}$ satisfies Equation (4) for the functions $\widetilde{E},\widetilde{T}$.

## Cauchy Functional Theorem

We provide a self-contained statement and proof of the version of the Cauchy functional theorem employed in Appendix A. The proof can also be found in standard textbooks; see, e.g., Theorem 3 in Aczel [7].

### Theorem 3

Let $H:\left( 0,\infty \right) \rightarrow \left( 0,\infty \right)$ be a function with the property that $H\left( xy\right) =H\left( x\right) \times H\left( y\right)$ for all $x,y\in \left( 0,\infty \right)$. Moreover, suppose *H* is continuous at least at a single point. Then there exists $\alpha \in \left( 0,\infty \right)$ such that for all $x\in \left( 0,\infty \right)$,

$$\begin{aligned} H\left( x\right) =x^{\alpha }. \end{aligned}$$

### Lemma 3

For all $x\in \left( 0,\infty \right)$ and any rational number *q*, $H\left( x^{q}\right) =H\left( x\right) ^{q}$ .

### Proof

Note that $H\left( 1\right) =H\left( 1\right) \times H\left( 1\right)$ which implies $H\left( 1\right) =1$. Moreover for any $k\in \mathbb {Z}$ with $k>0$ we have

$$\begin{aligned} H\left( x^{k}\right) =H\left( x\right) ^{k}. \end{aligned}$$

To extend this to $k\in \mathbb {Z}$ with $k<0$ note that

$$\begin{aligned} H\left( 1\right) =H\left( x\times \frac{1}{x}\right) =H\left( x\right) \times H\left( \frac{1}{x}\right) \Rightarrow H\left( x\right) =\left( H\left( \frac{1}{x}\right) \right) ^{-1}. \end{aligned}$$

Now let $x\in \left( 0,\infty \right)$ and $k,m\in \mathbb {Z}.$ Set $y=x^{1/m}$. Then

$$\begin{aligned} H\left( y^{m}\right) ^{1/m}=H\left( y\right) \Rightarrow H\left( x\right) ^{1/m}=H\left( x^{1/m}\right) . \end{aligned}$$

Hence we have

$$\begin{aligned} H\left( x^{k/m}\right) =H\left( x^{1/m}\right) ^{k}=H\left( x\right) ^{k/m}, \end{aligned}$$

as desired. $$\square $$

Let $S\subseteq \left( 0,\infty \right)$ be the set of all the rational powers of 2, that is $x\in S$ if and only if there exists $q\in \mathbb {\mathbb {Q}}$ such that $x=2^{q}$.

### Lemma 4

*S* is dense in $(0,\infty ).$

### Proof

Let $r,s\in (0,\infty )$ with $r<s$. We want to prove there is a rational number *q* such that $r<2^q<s$, which is equivalent to proving there is a rational *q* such that $r2^q<1<s2^q$.

Set $\alpha =\log _2 (1/r)$, so that

$$\begin{aligned} r2^\alpha =1<s 2^\alpha . \end{aligned}$$

Using the density of the rationals we can construct a rational sequence $q_n\rightarrow \alpha$ such that $q_n<\alpha$ for all *n*. Since the exponential function is continuous and strictly increasing, it follows that for large enough *N* we get

$$\begin{aligned} r2^{q_N}<1<s 2^{q_N}, \end{aligned}$$

as desired. $$\square $$

Now let $x\in S$, so that $x=2^{q}$ for some rational number *q*. By Lemma 3 we have

$$\begin{aligned} H\left( x\right) =H\left( 2\right) ^{q}. \end{aligned}$$

By definition of *x*, we know that for some $k,m\in \mathbb {Z}$, $q=k/m$. Therefore, $\frac{k}{m}=\log _{2}x=\frac{\log _{H\left( 2\right) }x}{\log _{H\left( 2\right) }2}$. It follows that

$$\begin{aligned} H\left( x\right) =H\left( 2\right) ^{\log _{H\left( 2\right) }\left( x\right) /\log _{H\left( 2\right) }\left( 2\right) }=x^{1/\log _{H\left( 2\right) }\left( 2\right) }. \end{aligned}$$

Setting $\alpha =1/\log _{H\left( 2\right) }\left( 2\right)$, we have proved $H\left( x\right) =x^{\alpha }$ for all $x\in S$.

Now suppose for contradiction there exists $z\in \left( 0,\infty \right)$ with $H\left( z\right) \ne z^{\alpha }$. Fix any $x,y\in S$. We will show for any $\varepsilon >0$ there exists a point $\left( x^{\prime },y^{\prime }\right) \in \left( 0,\infty \right) \times \left( 0,\infty \right)$ with $H\left( x^{\prime }\right) =y^{\prime }$, and $\left( x^{\prime },y^{\prime }\right)$ has Euclidean distance less than $\varepsilon$ from $\left( x,y\right)$. Hence for any $x,y\in S$, we can construct a sequence on the graph of *H* that approaches (*x*, *y*). Since *S* is dense in $\left( 0,\infty \right)$, we conclude that *H* is nowhere continuous on $\left( 0,\infty \right)$, which gives our contradiction.

To continue, define $\delta =H\left( z\right) /z^{\alpha }$, from which $\delta >0,\delta \ne 1$. Define $\beta \in \mathbb {R}$ to solve $x^{\alpha }=y/\delta ^{\beta }$. Such a $\beta$ exists because *y* and $x^{\alpha }$ are both non-negative. Now, for any $z^{\prime }\in S$ and $b\in \mathbb {R}$, define $x^{\prime }=x\left( z/z^{\prime }\right) ^{b}$. Using Lemma 3 we get

$$\begin{aligned} H\left( x^{\prime }\right) =H\left( xz^{b}z^{\prime -b}\right) =H\left( x\right) \times H\left( z^{b}\right) \times H\left( z^{\prime -b}\right) =H\left( x\right) \times \left( \frac{H\left( z\right) }{H\left( z^{\prime }\right) }\right) ^{b}. \end{aligned}$$

Given the definition of $\delta$, and using $x,z^\prime \in S$, we have

$$\begin{aligned} H\left( x^{\prime }\right) =x^{\alpha }\delta ^{b}\left( \frac{z}{z^{\prime }}\right) ^{\alpha b}. \end{aligned}$$

Applying the definition of $\beta$ yields

$$\begin{aligned} H\left( x^{\prime }\right) =\frac{y\delta ^{b}}{\delta ^{\beta }}\left( \frac{z}{z^{\prime }}\right) ^{\alpha b}=y\left( \frac{z}{z^{\prime }}\right) ^{\alpha b}\delta ^{b-\beta }. \end{aligned}$$

This equation holds for any choice of $z^{\prime },b\in S$. Now set $y^{\prime }=H\left( x^{\prime }\right)$. By choosing *b* very close to $\beta$ and $z^{\prime }$ very close to *z*, we can make $H\left( x^{\prime }\right)$ arbitrarily close to *y* and $x^{\prime }$ arbitrarily close to *x*, as desired.
